# Chemo-radiotherapy induced oral mucositis during IMRT for head and neck cancer - An assessment

**DOI:** 10.4317/medoral.20126

**Published:** 2015-02-07

**Authors:** Karthika Nagarajan

**Affiliations:** 1 M.D.S. Visiting Research Fellow (Honorary), Griffith Health Institute, Griffith University, Gold Coast, QLD, Australia

## Abstract

**Background:**

This study is conducted mainly to evaluate the changes in quality and quantity of oral epithelial cells during the course of IMRT.

**Material and Methods:**

30 Patients undergoing chemo-radiotherapy were followed through course of treatment. They were compared with a group of age- and sex-matched healthy individuals. The procedure involved WHO clinical scoring, collection of oral washings and preparation of buccal smears from both study group and control group. The changes occurred were recorded as a way of assessing the severity of oral mucositis.

**Results:**

Revealed a significant occurrence of oral mucositis in almost all patients during weekly follow up. There was a significant increase in percentage of viable buccal epithelial cells in study group when compared to normal controls (*P*<0.005) during and at the end of chemo-radiotherapy.

**Conclusions:**

Quantification of oral mucositis can be done at cellular level by determining the oral mucosal cell viability and their maturation during IMRT.

**Key words:**Oral mucositis, in vitro assay, quantification, radiotherapy, chemotherapy, viable cells.

## Introduction

Cytologic evaluation of irradiation effects on oral mucosa was reported in 1957 and on oral cancer in 1959 (1,2). Growing evidence indicates that more aggressive regimens improve local tumor control and survival of patients with head and neck cancer. These have come, however, at the expense of increased patient morbidity, notably an increase in severe mucositis that causes substantial pain, interferes with chewing, swallowing, and substantially worsens the patient’s quality of life (3).

Oral mucositis is a common, dose limiting and potentially serious complication of both radiation and chemotherapy. These therapies are nonspecific, interfering with the cellular homeostasis of both malignant and normal host cells. An important effect is the loss of rapidly proliferating epithelial cells in the oral cavity. Within the mouth, the loss of these cells leads to mucosal atrophy, necrosis and ulceration (4).

Oral mucositis induced by irradiation is defined as a reactive inflammatory-like process of the oral and oropharyngeal mucous membranes. The severity of mucositis is determined by the radiation parameters of dose per day, cumulative dose, volume of irradiated tissue and type of ionizing radiation (5).

Mucositis induced by antineoplastic drugs is an important, dose limiting and costly side effect of cancer chemotherapy (6). Direct toxicity to the oral epithelium is perhaps the most obvious drug induced cause. This usually occurs within 5 to 10 days post administration of medication. The drug induced neutropenia can manifest as mucositis (Indirect toxicity). Microbial culturing and PCR analysis are critical at this point to differentiate chemotherapy induced mucosal toxicity from mucosal neutropenic infectious complications caused by bacterial, fungal or viral microorganisms (7).

Radio-chemotherapy regimens induce high levels of acute toxicity, significantly higher than for radiotherapy alone. The addition of chemotherapy introduces systemic toxicity and can exacerbate local tissue reactions when used concurrently with radiotherapy. Mucositis is recognized as the principal limiting factor to further treatment intensification in such situations (8). As new agents become available and as combinations of radiotherapy and multiple drug chemotherapy are used concurrently, reports of apparent interaction are appearing frequently in the literature (9). Counting the percentage of viable oral epithelial cells in oral washings may be useful as an objective parameter in studies focused on mucositis prevention (10).

This study is aimed at quantification of oral mucositis that develops during the Intensity Modulated Radiation Therapy (IMRT) at the cellular level by determining the viability of oral mucosal epithelial cells and comparing them with clinical World Health Organization (WHO) grading through the period of therapy. The study also aims at proving the efficacy of this method in predicting mucositis at an earlier stage of IMRT when compared to the WHO clinical scoring which is being commonly used.

## Material and Methods

Participants: 30 patients who are diagnosed as having Head and Neck malignancy including salivary glands and undergoing chemo-radiotherapy as treatment option were selected and followed up through the course of treatment.

The control group consisted of age matched 30 normal healthy persons (16 male/ 14 female) who were systemically well and not under any medication or without any adverse habits. Exclusion criterias were: patients with any oral mucosal defect, patient who needs any obturator or prosthesis, treatment with antibiotics in the 2 week period before the start of therapy, oral candidiasis or acute periodontitis and patients with naso-gastric tube at the start of treatment.

The procedure involves clinical scoring, collection of oral washings and preparation of buccal smear from both study group and control group. For the study group, clinical procedures were done on the first day prior to the commencement of therapy. Then the next samples were taken at 7th, 14th and 21st day. If the patient experiences any complications, weekly samples will be collected till the oral mucosa returns to normal. Only the initial single samples were collected for healthy controls as per the inclusion and exclusion criteria. Proper Institutional Review Board (IRB) and Ethical committee approval were obtained from the Institutions before the start of the study. The patients also provided signed informed consent before the collection of initial samples.

- I. Oral mucositis scoring.

Patients who were to undergo chemoradiotherapy were clinically evaluated for mucositis and scoring will be done based on WHO scale. i.e. grade 0 - no change, grade 1 - soreness/ erythema, grade 2 - erythema/ ulcers/ can eat solids, grade 3 - ulcers/ requires liquid diet only, grade 4 - alimentation not possible (11). Buccal mucosa on the treated side and those regions of oral mucosae which were included in the Radiation Therapy (RT) target areas were included for the grading.

- II. Oral Washings.

Patients were asked to rinse (or gargle if the RT site was located in the posterior regions of the oral cavity) their mouth with 10ml sterile saline for 15 seconds and to spit into a glass beaker. This was centrifuged at 190g, 10 minutes at room temperature (ACSW-163 centrifuge machine, Atul chemicals and scientific works) and the centrifugate cells were obtained. The cells are suspended in 1ml of RPMI 1640 (Hi media Lab Pvt Ltd, Mumbai, India) medium containing fetal calf serum (Hi media Lab Pvt Ltd, Mumbai, India) 5% and the suspension is divided into two parts. A micro pipette (SC - single channel, Atul chemicals and scientific works) was used to obtain 50µl of suspension and was treated with 50µl try pan blue (Hi media Lab Pvt Ltd, Mumbai, India) - 0.4% and immediately transferred to haemocytometer and cell count was performed. The other part was incubated for 15 minutes with acridine orange (Hi media Lab Pvt Ltd, Mumbai, India) and diluted with phosphate buffer saline and were examined by fluorescence microscopy. The percentage of apoptotic buccal epithelial cells was determined. Cells were scored as apoptotic when their nucleus showed condensation.

- III. Buccal smear.

The buccal scrapings from representative sites (which were included in the RT plan) were selected which were expected to develop oral mucositis during the course. The sites were scrapped, fixed using ether alcohol after preparing the smear and stained using Papanicolaou stain. Epithelial cell morphology and differentiation were studied under the light microscope. Cells were graded as follows:

• Orange stained cells - mature

• Blue/green stained cells - immature

• Partly orange and partly green - intermediate maturation (12) 

Blood investigations reports were obtained from the patient records for the study group on the day of sample collection. Total count, differential counts were estimated and level of blood leukocytes and oral leukocytes were tabulated. Results were statistically evaluated using “t test”, Wilkoxson signed rank test.

## Results

The study group consisted of 30 persons who received chemo-radiotherapy by IMRT. The radiation doses received by the fourth week are between 999 rads to 4200 rads. The gender wise distribution consisted of 25 males (83%) and 5 females (17%). Major forms of carcinomas (CA) included were CA tongue (6 cases) followed by CA buccal mucosa (3), CA oropharynx (3) and CA tonsil (3) and also a case of mucoepidermoid carcinoma. Comparison of WHO mucositis grading from week 1 to 4 showed that there was a significant increase in incidence and severity of oral mucositis starting from week 2. A severe form of mucositis development was observed by the end of fourth week. In week 3 mean frequency of patients exhibiting grade 2 mucositis increased slightly and none of the patients were under grade 0 anymore. As the patients entered week 4 considerable numbers of patient exhibited grade 3 type of mucositis. Statistics showed *p*-value (< 0.0005) between week 1 and 2, week 1 and 3 and also week 1 and 4. The mean percentage viability between study and controls had a statistically insignificant difference. The test group showed statistically similar percentage of viable cells (*p*- 0.191) at the beginning of therapy as compared to the normal controls. There was a statistically significant increase in percentage viable cells from week 1 to week 4 (95% Confidence Interval - 1.628, 3.279, 3.400 and 4.29 respectively).

On comparing the cells of the buccal smears stained using PAP, there was an insignificant variation in the baseline difference between the mature cells of controls and week 1 sample of study group (*p* - 0.009) and a mild difference between intermediate cells (*p* - 0.013) of the study group and control while the percentage of immature cells (*p* - 0.805) was almost similar. Mature, intermediate and immature cells were compared in study group during treatment. A statistically significant (*p* - <0.0005) decrease in percentage of mature cells from week 1 to week 4 was observed and intermediate cells showed insignificant variations ( *p* - 0.129, 0.081 and 0.243) for weeks 2, 3 and 4 respectively. Immature buccal cells showed statistically significant (*p* < 0.0005) increase from week 1 to week 4. The WHO mucositis score corresponded with viable cell count and showed earlier change when compared to WHO grading. There was no statistically significant difference between patients who underwent only radiotherapy with IMRT and who underwent adjutant chemo-radiotherapy (*P*-0.184) in percentage of viable cells.

## Discussion

Complications associated with chemo-radiotherapy can be direct, caused by toxic action of treatment agents on the proliferative mucosal lining of the mouth or indirect, the result of hemopoeitic shut down (13).The earliest signs and symptoms of oral mucositis include erythema and edema, a burning sensation, and an increased sensitivity to hot or spicy food. (14).The grade I to grade IV mucositis can be evaluated on clinical grounds (15,16).

In our present study the WHO mucositis clinical grading system; which was demonstrated to very efficiently represent the clinical scale in accordance with a validated questionnaire was utilized (17). This showed that every patient had a significant increase in the gradation of mucositis mostly starting from the 2nd week of treatment and by the end of the 4th week, grade III or grade IV mucositis. There was a statistically significant difference between the clinical grade of mucositis between week 1 and week 2. In order to overcome the disadvantages of clinical scoring system, we used the in vitro assay (10) that showed increase in viable oral epithelial cells which correlated with the increase in number of immature epithelial cells. This was believed to be due to an increased desquamation of the upper oral epithelial layers after high-dose chemotherapy.

**Figure 1:**
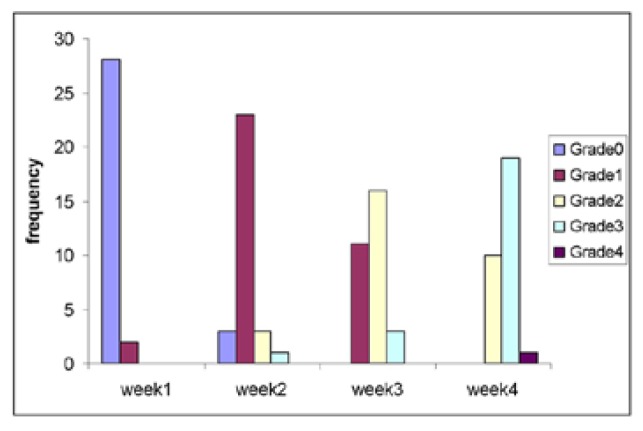
Frequency table comparing the incidence of oral mucositis grading from week 1 to week 4.

The main reason for undertaking the study is to estimate whether the state of art IMRT has any advantage over conventional RT. In our study, approximately 63% of patients developed severe form of mucositis as per the WHO mucositis grading scale (Fig. 1). The mean percentage viability difference between pretreatment study and normal controls were (*p*-0.191). Increasing percentage of viable epithelial cells ( Table 1) can be considered an earlier indicator of development of mucositis (10,18). The Confidence Interval (95%) compared from week 1, 2, 3 and 4 (1.628, 3.279, 3.400, 4.29) did not exhibit any overlap evidencing the statistical significance of using viable cell percentage for predicting the mucositis development ( Table 2).

**Table 1:**
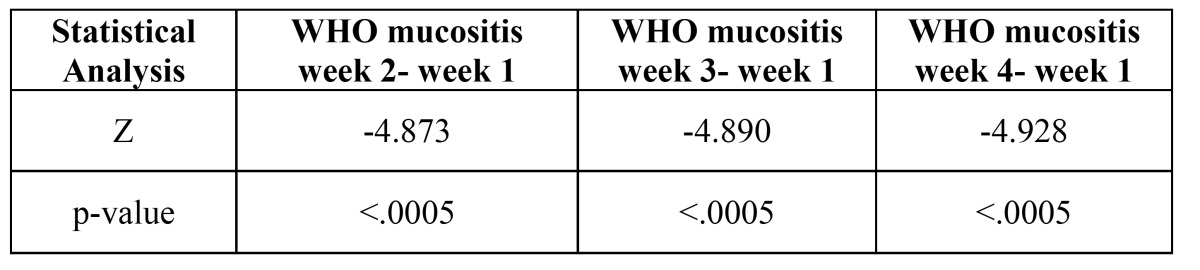
The Wilcoxon signed rank test comparing WHO mucositis grading.

**Table 2:**
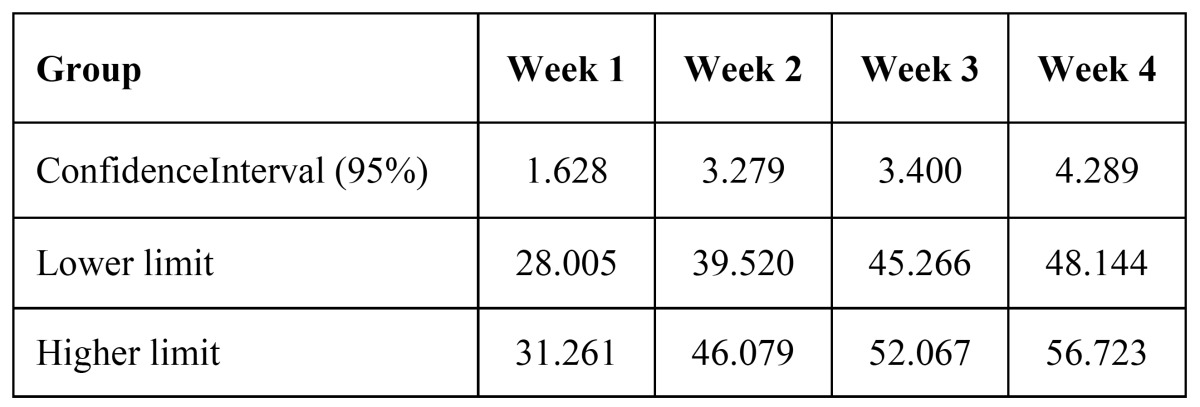
Confidence Interval (95%) of viable cells from week 1 to week 4.

In the present study of chemo-radiotherapy, the viable epithelial cell levels were compared using the Pap stained buccal smears by counting the percentage of mature, intermediate and immature cells. The study group and control group showed statistically insignificant differences in intermediate and immature cells (*p*-0.013, 0.805) whereas, a significant difference was observed in mature cells (*p*-0 .009) during the start of therapy. On progression of treatment; mature cells showed a statistically significant *p* (<0.0005) decrease starting from week 2 to week 4. While the immature cells showed a significant increase from week 2 to week 4, the mean difference being 6. 2, 9.9 and 13.4 respectively. The intermediate type of cells appeared to be in constant number as can be observed from the p value of 0.129, 0.081, 0.243 which showed statistically insignificant difference. The increase in percentage of immature cells corresponded to the increase in viable epithelial cells. This phenomenon was probably due to an increased desquamation of the upper oral epithelial layer after high dose chemotherapy (10).

**Figure 2:**
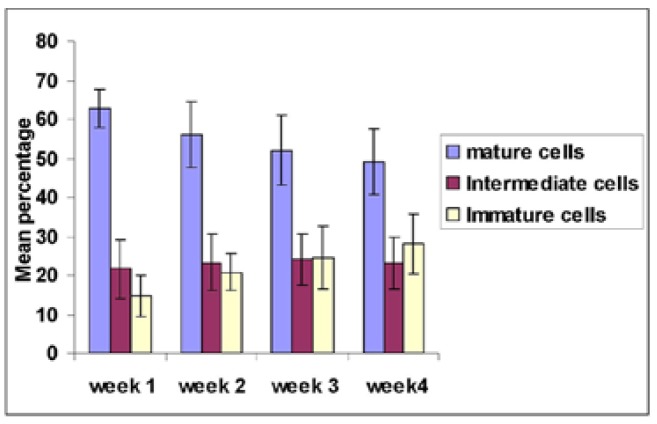
Comparison of mature, intermediate and immature buccal cells between week 1, week 2, week 3 and week 4 of study group.

**Figure 3:**
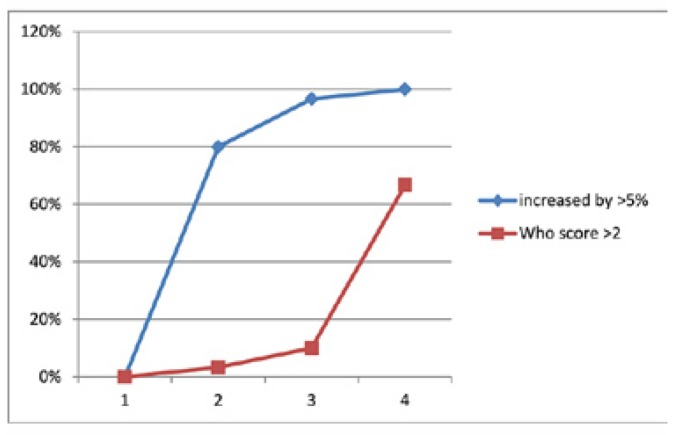
Comparison of Viability percent and WHO score from week 1 to week 4 – shows viability. showing marked increase during week 2 and earlier than WHO score as a better predictor of Oral mucositis occurrence.

The chemo-radiotherapy induced oral mucositis has been extensively evaluated in patients receiving Hematopoietic Stem Cell transplantation (HSCT) and a study by Archibald *et al*. state that the addition of chemotherapy to the treatment regimen did not increase the incidence of complications when compared with historical controls receiving radiotherapy alone (19,20). Our study using IMRT also showed a similar pattern of behavior of upper epithelial cell layer (Fig. 2). Our study showed a shift from mature to immature cells (10) and this proves the profound effect of chemotherapy on cells when compared to radiotherapy. There was statistically no difference in this trend between the patients who underwent chemo-radiotherapy and radiotherapy alone (18). The findings also correlated well with the increased percentage of viable epithelial cells as the chemo-radiotherapy progressed. There was a significant increase in viable cells as compared to the WHO score in second week which is also well evidenced by increase in number of mature cells by the end of week 4 (Fig. 2). The change in viability preceded the change in WHO score (Fig. 3). This means that this assay is more sensitive for the detection of mucositis in adjunct to the clinical WHO toxicity grading system.

In conclusion, after high-dose chemoradiotherapy, the percentage of viable oral epithelial cells increases. Also, a shift from mature to immature cells in the buccal epithelium is observed. This is possibly due to a desquamation of the upper oral epithelial layer. The specific target calculation during IMRT can reduce the total area of the oral mucosa affected due to chemoradiotherapy but will not be significant. Newer treatment modalities can be considered that can counteract the side effects of chemotherapeutic agents. A larger clinical trial would be giving more accurate results in this regard. A decreased loco-regional control, poorer quality of life and shortened overall survival has been recently associated with unplanned treatment breaks and reduction in dose intensity (21). Therefore such assessment aids in mucositis can become valuable in future. The in-vitro assay utilized may also be useful as an adjunct in studies focused on oral mucositis prevention.
